# Suicide

**Published:** 1885-04

**Authors:** 


SUICIDE.

The last necrological report of the Illinois State Board of Health, gives the reported cause of death of 202 physicians. Six deaths, about three per cent. of the entire number, have been attributed to suicide. A very large percentage as compared with other occupations. Again, we have five deaths in which the reported cause of death is an "overdose of morphia," and in two others an "overdose of chloral." It does not seem probable that these "overdoses" were all accidental. If we did so regard them, we must admit that a large number of deaths are caused by the careless use of two powerful sedatives, for it is not supposable that physicians will only make mistakes when prescribing for themselves. Again, it would be very singular to have the mistakes confined strictly to the sedative poisons, to the entire neglect of those acids and powerful substances which cause a more or less painful death. The law of chance is not so beneficent in its operations. We cannot but class many of these cases among the suicides, and regard the "overdose" statement as a delicate shield placed there more for the comfort of relatives than out of strict regard for the truth.

